# PRIME-BSPre: a genome-wide protein-RNA binding sites prediction method based on templates

**DOI:** 10.1186/s12864-026-12657-3

**Published:** 2026-02-21

**Authors:** Xinhang Wei, Yingtian Duan, Danyang Li, Xudong Liu, Juan Xie, Shiyong Liu

**Affiliations:** School of Physics, Huazhong University of Science and Technology, Wuhan, Hubei 430074 China

**Keywords:** Protein–RNA interaction, RNA-binding proteins (RBPs), Binding site prediction, eCLIP, Template-based method, RNA secondary structure, Computational genomics, Transcriptome-wide analysis

## Abstract

In this paper, we present PRIME-BSPre, a template-based genome-wide method for predicting protein-RNA binding sites that incorporates the RNA sequence and secondary structure as well as the tertiary structure of corresponding RBPs. We are pioneers in introducing low Shannon entropy algorithm in PRIME-BSPre to describe the binding preferences of RBPs on RNA motifs. The LS-PEAK derived from LS-Scores in PRIME-BSPre is utilized to optimize the alignments screening. PRIME-BSPre has been successfully benchmarked on the human genome, demonstrating its excellent prediction performance on independent RBP datasets and its robustness across different cell lines.

**Supplementary Information:**

The online version contains supplementary material available at 10.1186/s12864-026-12657-3.

## Introduction

Post-transcriptional regulation is a crucial aspect of gene expression, and RNA-binding proteins (RBPs) play key roles in this process [1, 2]. To fully comprehend the physiological functions and potential contributions of RBPs, it is imperative to identify their interacting RNAs and specific binding sites [3]. Enhanced CLIP (eCLIP) is an in vivo method derived from CLIP-seq (crosslinking and immunoprecipitation followed by sequencing), which enables the study of protein-RNA interaction on a genomic scale [4]. The eCLIP output data have demonstrated utility in investigating the functional roles and binding preferences of RBPs [3, 5, 6], and are widely used to predict possible protein-RNA interactions [7, 8]. Compared to iCLIP [9] and PAR-CLIP [10], eCLIP offers the advantages of simplicity, high success rate, high resolution, and partially addresses the issue of biased base composition of RNA sequences caused by cellular factors. And in comparison, classical in vitro method such as SELEX [11], RNAcompete [12] and RNA Bind-n-Seq [13] which were developed subsequently, eCLIP is more physiologically relevant and better restores the native structure of RNAs due to its cellular context. Additionally, the nucleotide sequences utilized in eCLIP are more comprehensive and representative of their natural state compared to synthetic sequences used in vitro that were fragmented prior to interacting with RBPs.

Faced with a large number of RBPs and their corresponding RNAs, experimental research alone has its limitation. Thus, many prediction models for protein-RNA interaction have been proposed on the basic of both in vivo and in vitro sequencing data. Some classical computational methods, such as free docking and template-based approaches, were used to investigate protein-RNA interactions. The template-based approach is based on the hypothesis that similar protein sequences may fold into similar structures [14]. Based on this hypothesis, the template-based prediction method PRIME was proposed to predict the protein-RNA complex interactions [15]. The accuracy of template-based models depends on the quality of the selected template, as determined by sequence or structural alignment. In the last two decades, most prediction methods rely on deep learning models or machine learning techniques were proposed, such as RCK [16] and GraphProt [17]. However, training these models requires high-confidence, low-noise and well-structured data. Therefore, researchers prefer to train their models using RNAcompete and RNA Bind-n-Seq datasets rather than CLIP-seq. And this may lead to following problems: (1) As previously stated, the in vitro experimental methods utilize synthetic RNA sequence fragments that lack complete secondary structure during binding, thus preventing the acquisition of structural preferences from the data. So certain methods such as RNAcontext [18] or PrismNet [7] choose to use prediction methods such as Sfold [19] or experimental data obtained from icSHAPE [20] to supplement the absence of RNA secondary structure in their datasets. PrismNet [7], a newly launched server for predicting protein-RNA binding sites, outperforms other mainstream deep learning methods and also incorporates RNA sequence and secondary structure characteristics. However, the supplemented data cannot fully capture the actual binding process, potentially resulting in a discrepancy between sequence-based and structure-based binding preferences learned by models. (2) The binding preference obtained by the aforementioned experimental methods is heavily reliant on RBPs. In other words, each deep learning model can only be trained and accurately run prediction under one specific RBP.

Typically, the features observed at RNA binding sites can be classified into two distinct categories: sequence-based and structure-based. Obtaining specific features of the binding sites is crucial for accurately predicting protein-RNA interactions and identifying the strength of their binding affinity. Earlier methods, such as DeepBind [21], solely relied on sequence-based information to train models and achieved some success. However, approaches like GraphProt [17] and RNAcontext [18] have incorporated RNA secondary structure features [22] into their models, resulting in improved performance. In terms of selecting RNA secondary structure features, most regression models [8] or sequence-based neural network models only consider the pairing information [19]. While some methods serialize the complete secondary structure, a few methods, such as GraphProt, employ graph networks to directly extract both the secondary structural components and their corresponding sequence information. However, as mentioned above, these deep learning approaches have their limitations on prediction. Each specific deep learning model can only be used to predict the binding of corresponding RBP trained under corresponding cellular environment. These restrictions make these deep learning approaches unable to predict all binding sites and make it difficult to predict on the models that have not been trained.

Moreover, investigations into the binding preferences of RBPs have revealed a remarkable conservation in their RNA binding domains and the specific regions of RNAs they interact with [23, 24]. Therefore, we aim to develop a universal prediction model based on these characteristics, considering these binding preferences of RBPs. As such, template-based prediction method PRIME-3D2D has been proposed in previous research [25]. This method can basically complete the prediction on the yeast genome, but its template scoring function only considers the global similarity between RBPs and RNAs, ignoring the local characteristics of binding sites. In this study, PRIME-BSPre is introduced to expand the template-based prediction method. Its alignment and template screening process incorporates both the RNA sequences, secondary structures, and the corresponding tertiary structures of RBPs (see Materials and methods). And it has been successfully benchmarked on the human genome.

## Materials and methods

### Template library construction

Template library constructed here contains 158 human RBPs and 15,348 protein-coding transcript entries (mRNAs; CDS sequences) that interact with these RBPs (see Supplementary Table S1). Supplementary Table S1 lists all RBPs in the library, the number of template RNAs per RBP, and the corresponding eCLIP cell line(s) used. Each RBP has multiple binding RNAs in the template library, and the library provides a total of 15,348 RBP–RNA pairs for template alignment. In contrast to the studies focusing solely on RNA fragments, our templates accurately map the eCLIP data across the entire genome, and all templates are built based on full-length coding sequences (CDS) of protein-coding transcripts. To obtain the RNA binding sites locations of human RBPs, eCLIP-bed files are downloaded from ENCODE [26] (https://www.encodeproject.org/) and are cross-referenced with CDS files of human transcripts obtained from Ensembl database [27] (http://www.ensembl.org/index.html). For each transcript entry, only eCLIP peaks fully contained within the CDS genomic interval are retained to construct the binding-site mask; therefore peaks located in 5′UTR, 3′UTR, intronic regions, and noncoding transcripts are excluded. The retained/excluded peak counts after this CDS restriction (and the 2000-nt length filter) are summarized in Supplementary Table S4. Accordingly, non-coding RNAs (e.g., lncRNAs and small non-coding RNAs such as tRNAs and rRNAs) are outside the scope of the current library and test sets.

Using SAMtools [28], complete transcript sequences are extracted from the HUMAN hg38 genome assembly [29] (https://hgdownload.soe.ucsc.edu/downloads.html). In addition to considering RNA sequence similarity, the algorithm in PRIME-BSPre incorporates the secondary structures of interacting RNAs and the tertiary structures of RBPs. RNAstructure [30] is utilized for predicting RNA secondary structures. Due to limitations in precision and computational time, the maximum allowed transcript length was restricted to less than 2000nt. We quantified the effect of this cutoff on the candidate set used in template construction, defined as transcripts containing at least one eCLIP peak fully contained within the CDS interval. Under this definition, 70,530 out of 72,861 candidate transcripts (96.80%) were excluded by the > 2000 nt filter, leaving 2,331 transcripts (≤ 2000 nt) for downstream template library construction. The template library is organized as RBP–RNA pairs to enable RBP-specific template alignment. As a consequence, the same transcript may appear in the library under multiple RBPs, which reflects shared eCLIP-supported binding evidence across RBPs rather than duplicated counting. In the final template library, we obtained 15,348 RBP–RNA pairs comprising 2,455 unique transcripts; among them, 1,971 transcripts (80.29%) are associated with at least two RBPs (maximum 42 RBPs per transcript) (Supplementary Table S4).

PDB files are obtained primarily for 158 RBPs from experimental data available on UniProt (https://www.uniprot.org/). Proteins with incomplete experimental structures are supplemented with predicted results from AlphaFold Protein Structure Database [31, 32] (https://alphafold.com/). When AlphaFold models are used, we do not explicitly filter by pLDDT; structural similarity is assessed by TM-align based on the residues that can be reliably superposed, and this may be less accurate for highly disordered proteins; consequently, poorly modeled/disordered segments generally do not improve the alignment score and may even reduce structural similarity, making such templates less likely to be selected.

### Test sets construction

Test set 1. To assess PRIME-BSPre, binding data from different CLIP experiments that performed on different cell lines (the cell line HepG2 and the cell line HEK293) [7, 33–35] are collected to prepare Test set 1. Accordingly, Test set 1 applies the same CDS-based filtering described above, and only peaks fully contained within Ensembl CDS intervals are used to define binding-site masks. The Test set 1 is independent of the template library since both the RBPs and the protein-RNA binding data are different from the templates. The construction of Test set 1 follows the same process as that of the template library. Test set 1 finally contains 9 RBPs (PRPF39, NF90, PABPN1, TIAL1, ZC3H8, C17ORF85, ALKBH5, CAPRIN1, C22ORF28) and corresponding 665 binding RNAs on these RBPs.

Test set 2. Test set 2 is constructed from the template library to evaluate the predictive ability of PRIME-BSPre. RNAs with a minimum Irreproducibility Discovery Rate (IDR) [36] for each RBP in template library is chosen. In general, IDR is used to describe the reproducibility of the replicates and thus can indicate the validity of experiments [37]. In the eCLIP-seq Processing Pipeline v2.0 [26], peak calling, IDR and signal normalization are implemented to obtain significant binding peaks, and IDR is used to filter peaks from all control experiments to obtain the final binding signals. Ultimately, RBP-RNA strong binding pairs for all the RBPs in template library are finally selected and comprise Test set 2. Because Test set 2 is derived from the template library, it inherits the same CDS-restricted scope.

### Workflow of PRIME-BSPre

**Fig. 1:**
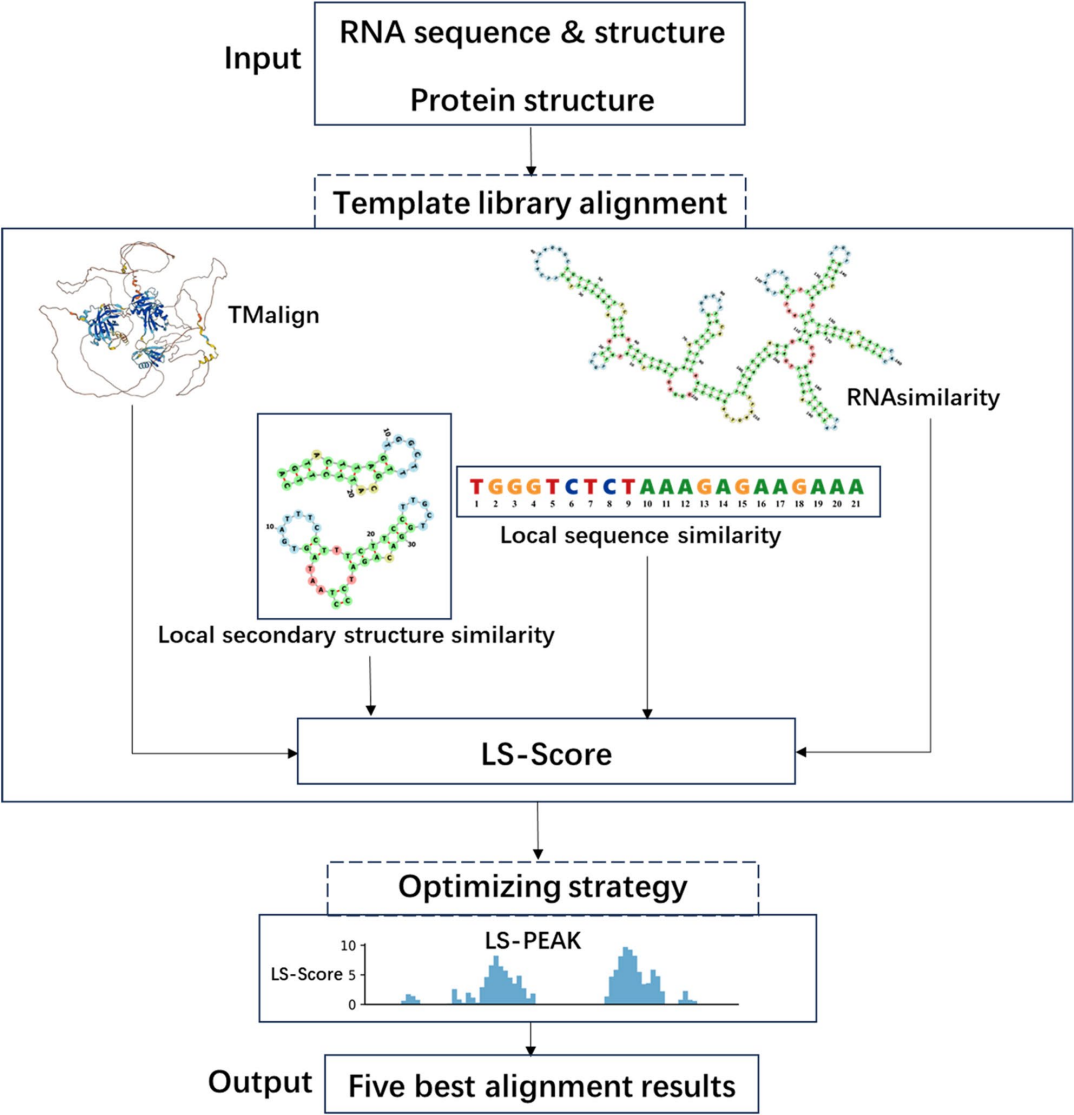
Workflow of PRIME-BSPre. The input target is aligned with all the templates in template library. Tertiary structure similarity of RBPs, binding preference of binding sites, global and local similarity of RNA sequences and secondary structure are considered in PRIME-BSPre scoring methods. The alignment results are initially ranked by the LS-Scores and five best alignments are finally selected by the LS-PEAK as the prediction results

Figure 1 shows the workflow of PRIME-BSPre. The sequence and secondary structure of the RNA, as well as the PDB file of queried RBP are required in template alignment. These inputs are utilized to align against the entire template library. TMalign [38] is used to align the tertiary structure of the target protein with the RBPs in the template library, yielding a similarity score (TM-Score). The sequence and secondary structure of the target RNA are primarily aligned with RNAs in library by LocARNA [39] to give RNAsimilarity between the target and the template. The low entropy scoring function, local similarity scoring function and LS-PEAK optimizing strategy in PRIME-BSPre are designed to optimize the selection of templates as well as the alignment results with templates. The low entropy scoring function is designed to filter the templates with considering the binding preference of RBPs on RNA motifs. Local similarity scoring function incorporating local similarity of RNA sequence and secondary structure identifies precise features on binding sites. Local similarity score (LS-Score) for each prediction result is given after alignment, and all the prediction results are then pre-ranked by LS-Score. Five best alignment results are finally selected as the final prediction outputs through the LS-PEAK optimizing strategy.

### Runtime and resource profiling

To quantify practical computational requirements, we profiled PRIME-BSPre with stage-wise logging of wall-clock time, peak resident memory (RSS), and parallel worker usage. Profiling was performed using a lightweight psutil-based monitor that samples the main process and its child processes at a fixed interval during each pipeline stage (alignment, pruning, scoring, and LS-PEAK post-processing). We report stage-wise wall time and peak RSS, and the execution environment to support reproducibility and practical deployment planning. The resulting measurements and environment specifications are summarized in Table S5.

### Low entropy scoring function and binding preferences of RBPs

In PRIME-BSPre, binding preferences of RBPs on RNA motifs are considered and the low entropy scoring function is initially designed to improve the efficiency of selecting templates in alignment. Binding motifs in template binding regions are obtained through a sliding window. The length of the sliding window is 8 mer, with a sliding step of 6nt. The Shannon entropy of a binding motif (8 mer) is calculated as follows:

$$Shannon\;entropy\;of\;motif=\sum\limits_{i=A,C,G,T}-\left(P_i\ast\log_2P_1\right),P_1=\frac{N_i}8,$$

where $\:i$ is one of the bases A、C、G、T in transcript, $\:\:{N}_{i}\:$is the number of $\:i$ in 8 mer motif and $\:{P}_{i}$ is the probability of each base appearing in the motif. Motif with Shannon entropy lower than 1.1 in these 8mer motifs is defined as low entropy motif, and the lower the complexity of the motif the lower its Shannon entropy is. Consistently, the retained low-entropy motifs frequently exhibit simple-repeat-like low-complexity patterns (e.g., short homopolymers or di-/tri-nucleotide repeats), as summarized in Supplementary Fig. S1.

We set the sliding-window length to 8 nt to match the typical short-motif length scale of sequence-specific RBP recognition elements, which is commonly represented using short k-mers (often in the 4–8 nt region) in large-scale RBP specificity mapping and motif compendia. Choosing the upper end (8 nt) increases specificity while remaining within a stable, well-sampled k-mer region for transcriptome-scale scanning. We set the step size to 6 nt to reduce redundancy and correlation inflation across adjacent windows (stride 1 would share 7/8 positions), while maintaining dense local coverage (2-nt overlap). Finally, we define low-complexity motifs using a Shannon entropy cutoff of H < 1.1 bits: H = 1.0 corresponds to an exact two-nucleotide composition and H = 1.1 corresponds to an effective alphabet size of $\:{2}^{1.1}\approx\:2.14$, thereby capturing the single-base-rich and AU-/CU-rich compositional motif classes reported to be enriched in human RBP binding preferences.

### Local similarity scoring function for template library alignment

Despite primly aligned with templates, most predicted binding sites are found to be inaccurate when compared to the native binding sites; only a few predictions are deemed highly accurate. The original 3D2D-Score $\:(W*T{M}_{score}+（1-W）*RNAsimilarity,\:\:W=0.6)$ [25] in alignment with templates had a poor screening capability, and only a limited number of effective templates could be identified. 3D2D-Score was constructed for protein-RNA complexes; both TM-Score and RNAsimilarity are global. The 3D2D-score was deemed competent for protein-RNA complex analysis due to the RNA sequences in the complexes being generally short. However, when aiming to identify precise locations of the binding sites within longer RNA sequences, the 3D2D-score alone is proved to be insufficient, necessitating the development of the Local Similarity score (LS-Score). The local similarity scoring function is a scoring function specifically designed for the assessment of similarity between native binding sites on templates and their corresponding predicted binding sites on target RNAs. It comprises two main components, namely LS-2d (local secondary structure similarity) and LS-seq (local sequence binding preference similarity).

**Fig. 2:**
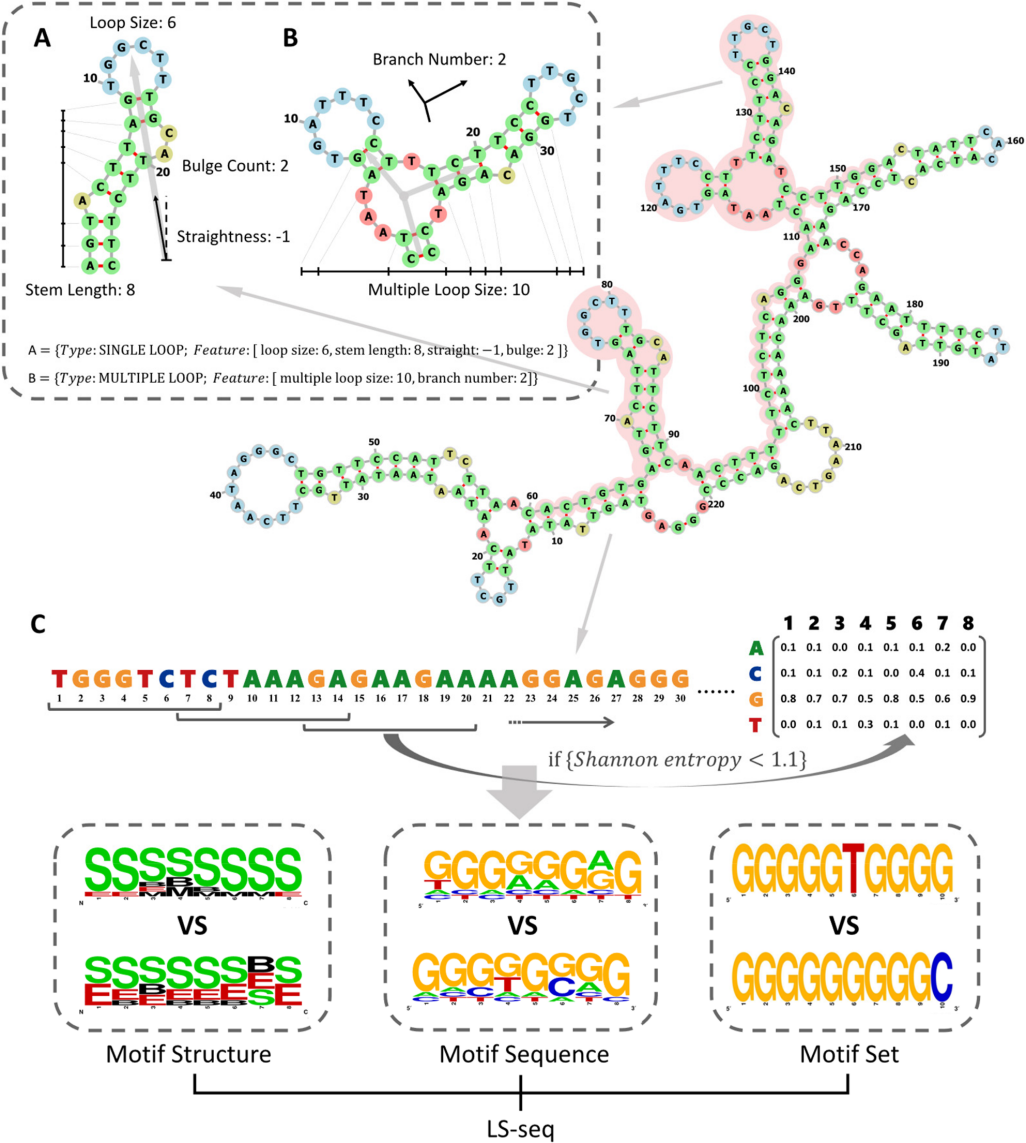
Local secondary structure and sequence features extracted on RNA binding sites. The pink-shaded region in the RNA secondary structure represents the binding sites, while LS-Score quantitatively assesses sequence and secondary structure similarities within this area between templates and targets. **A** The secondary structure HAIRPIN LOOP, which exhibits typical characteristics, is depicted along with its quantitative features. **B** The secondary structure MULTIPLE LOOP, which exhibits another characteristic feature, is depicted along with its corresponding quantitative attributes. **C** shows the Sequence features of native and predicted RNA binding sites: Motif Structure, Motif Sequence and Motif Set. Among them, Motif Structure is the Position Probability Matrices (PPMs) generated by serialized secondary structure features: B (Bulge), E (External strand), H (Hairpin loop), M (Multiple loop) and S (Stem); Motif set is a sequential description of all binding preference motifs, where each base represents the base enriched in motifs

LS-2d (Local secondary structure similarity). Preliminary alignment may be influenced by strong similarity regions outside the native binding sites, resulting in the actual similarity of the binding sites not being that strong. Thus, LS-2d approach is developed to extract, quantify and compare the characteristic secondary structures between the native and predicted binding sites. The local characteristic secondary structures are categorized into two groups: SINGLE LOOP and MULTIPLE LOOP (Fig. 2A and B). SINGLE LOOP includes loop size, stem length (the number of paired bases in the stem), bulge count (the number of bulges present on the stem), and straightness (by calculating the difference between the size and number of bulges on both sides of the stem, the distribution characteristics of bulges are described). Due to the complexity of MULTIPLE LOOP, we mainly focus on two key features: branch number and multiple loop size, and analyze the RNA secondary structures presented in dotted-bracket format. The similarity score is computed separately for each quantified featureand subsequently integrated into the LS-2d metric. This method systematically explores the characteristic secondary structures of both the target and template, selecting the maximum LS-2d value as the final matching outcome.

LS-seq (Local sequence binding preference similarity). In contrast to the traditional similarity score obtained through sequence alignment, an 8-mer sliding window with a step size of 6nt is utilized to scan both native and predicted binding sites. During this process, motif structure, motif sequence, and motif set are extracted and a quantitative analysis of these three features is conducted. The study of Daniel et al. on binding preference of human RBPs revealed that RBPs tend to bind RNA motifs with low complexity and large number of motifs with strong binding preferences were abundant in the single-base-rich and A/U-rich or C/U-rich regions [23]. Based on this finding, Shannon entropy of each motif in the sliding window is calculated, and motifs with Shannon entropy less than 1.1 are referred to as binding preference motifs (Fig. 2C, Shannon entropy of motif < 1.1). Subsequently, Position Probability Matrices (PPMs) based on the secondary structure and sequence of these selected binding preference motifs are constructed. To facilitate PPM description, secondary structure features are categorized into five units: B (Bulge), E (External strand), H (Hairpin loop), M (Multiple loop), and S (Stem). Furthermore, for each selected binding preference motif, it was named after the base with the highest count to generate a set of all motifs (Fig. 2C).

### LS-PEAK optimizing strategy based on the LS-Score

However, prediction results utilizing only LS-Score as a filter for Top5 alignments with templates are not good, some of the predictions on Top 5 alignments ranked by LS-Scores give low Matthews Correlation Coefficient (MCC) when mapped to the native binding sites. To investigate the underlying reasons and take statistical information of LS-Score into account, the following procedures are implemented for each alignment. The goal is to identify 8-mer RNA motifs on the target sequence that exhibit strong binding preferences and significant sequence similarity with native binding sites. Therefore, LS-Scores of all alignment results are considered, rather than only focusing on Top 5 results. LS-PEAK is generated to select the final outputs.

**Fig. 3:**
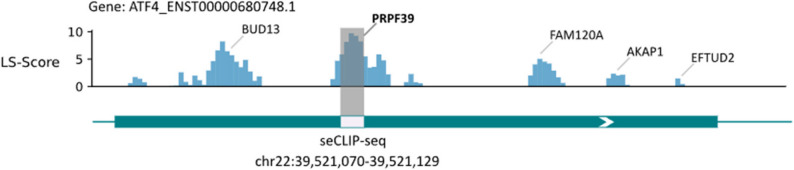
LS-PEAK on ATF4_ENST00000680748.1 with native seCLIP-seq binding sites. The upper blue histogram shows the base-resolution LS-Score along ATF4_ENST00000680748.1, where the height of each blue bar represents the LS-Score at the corresponding nucleotide position (higher bars indicate higher inferred binding preference). The gray shaded vertical window indicates the LS-PEAK region selected in this example: it is defined as a contiguous segment where LS-Scores exceed the mean LS-Score of this transcript (used here as the cutoff), and it corresponds to the candidate region carried forward for template screening. Protein labels above the histogram (e.g., PRPF39, BUD13) mark the RBPs whose native binding sites are shown for comparison at the corresponding genomic positions. The lower track displays the seCLIP-seq-supported native binding site intervals on the same transcript/locus (shown as the labeled binding segment aligned to the coordinate axis), enabling direct visual comparison between the predicted LS-PEAK window (gray shaded) and the experimentally observed binding locations

For each prediction result, LS-Score is evenly distributed to the bases ($\:v$) of motifs screened by low Shannon entropy algorithm at the predicted binding sites to get a set of the LS-Score for per base on the low entropy motifs:

$$S=\left\{v\vert v=\frac{score}{8\times n},\left(score,n\right)\in\left\{\left(LS_{score},N_{motif}\right)\right\}\right\}$$

where $\:v$ is the score for each base on predicted motifs of target RNA, and $\:S$ is a set of score $\:v.\:\:{LS}_{score}$ is the local similarity score of target RNA, and $\:{N}_{motifs}$ is the number of motifs on target RNA. Then, $\:v$ for all the prediction results are accumulated to calculate a $\:{Value}_{i}$ for each base on the target RNA sequence:

$$\:{Value}_{i}={\sum\:}_{v\in\:S}v$$

where $\:i$ is the base on target RNA and $\:{Value}_{i}$ is the accumulated score for each base. LS-PEAK for target RNA is generated subsequently utilizing these Values, revealing binding preference for RBPs. To summarize, LS-PEAK is a statistical peak map under base-resolution. In general, in the eCLIP data, it is observed that not only the general phenomenon that different binding sites being associated with distinct RBPs, but also numerous instances where multiple regions on a single transcript bound to the same RBP and different RBPs interacted with the same location on one transcript. Thus, in LS-PEAK, more regions are selected as potential strong binding preference regions. Top 100 predicted binding sites ranked by LS-Score are first selected, and the distance from their midpoint to five highest peaks on LS-PEAK map is calculated. Five predicted binding sites closest to these five peaks are finally selected as the output of the results. In the prediction of binding sites on ATF4_ENST00000680748.1 (ATF4 binding RNA) interacting with the protein PRPF39 in Fig. 3, the mean value of all LS-Scores was used as the threshold to intercept LS-PEAK, and some separate peaks were kept. The main peak was experimentally demonstrated to bind to PRPF39, and the sub-peaks were also experimentally proven to be native binding sites that interact with other proteins.

### Low-entropy motif screening and binding-preference summarization

We screened low-entropy motifs within template-library binding regions using Shannon entropy computed over a sliding window. Specifically, we applied an 8-nt sliding window with a 6-nt step across each binding region and retained windows whose Shannon entropy was below the predefined cutoff (low-entropy motifs). To summarize sequence-level preferences, we aggregated nucleotide composition across all retained low-entropy motifs and reported enriched base patterns at the cohort level (Supplementary Fig. S1B). To summarize structure-level preferences, we represented the predicted secondary structure of each low-entropy motif using five structural units (B: bulge, E: external strand, H: hairpin loop, M: multi-loop, S: stem). A motif was defined as “structure-unit–rich” if the length contributed by a single unit was at least half of the motif length (≥ 4 nt for an 8-mer); otherwise, it was categorized as “multi-structure.” We then quantified the distribution of these categories across all low-entropy motifs (Supplementary Fig. S1C).

To assess positional binding preference along transcripts, each transcript was normalized into 10 equal-length relative bins, and binding-site occurrences were mapped to bins and aggregated across RBPs to obtain a cohort-level positional distribution (Supplementary Fig. S1D).

### Ablation evaluation of LS-2d, LS-seq, and LS-PEAK

To quantitatively substantiate the contributions of LS-2d, LS-seq, and the LS-PEAK selection strategy, we performed a strict ablation study that follows the same execution pathway used in the default pipeline. Using the same set of candidate templates generated in the scoring stage, we evaluated four conditions: (1) FULL, i.e., the default setting where LS-Score is computed with all terms enabled and final candidates are selected by LS-PEAK; (2) –LS-2d, where the LS-2d term is set to zero during LS-Score recomputation, followed by re-running the profiling/peak-detection step and LS-PEAK selection; (3) –LS-seq, where the LS-seq term is set to zero during LS-Score recomputation, followed by re-running profiling/peak detection and LS-PEAK selection; and (4) –LS-PEAK, where LS-PEAK is disabled and candidates are directly ranked by LS-Score. Detailed per-pair results are provided in Supplementary Table S3.

## Results

PRIME-BSPre is benchmarked on different cell lines on Test set 1 and Test set 2. In addition, the prediction ability across different cell lines and the effectiveness of low Shannon entropy feature in the scoring function of PRIME-BSPre are compared with other methods. In comparison with PrismNet, it shows that when the PrismNet model trained on the HepG2 cell line used to predict the same RBPs on the HEK293 cell line, the prediction performance dropped significantly. This may be the fact that during training, the model learns some specific features caused by cell lines, experimental methods and environment except for the binding preferences, which may further reduce the universality of the model. However, PRIME-BSPre still achieves a robust prediction ability across different cell lines.

### Benchmarking of PRIME-BSPre

PRIME-BSPre is benchmarked on Test set 1. Table 1 shows the overall prediction results of PRIME-BSPre, PRIME-3D2D and PrismNet on cell line HepG2 and cell line HEK293 in Test set 1. PRIME-BSPre achieves the highest MCC on both two cell lines. Since PrismNet did not train models for RBPs, the prediction results of PrismNet are not good. We also report ACC in Table 1 for completeness and consistency with prior studies; however, ACC can be inflated in nucleotide-level binding-site prediction because true negatives dominate the full-length transcript background when predicted binding sites are much shorter than the CDS. Therefore, we interpret performance primarily using MCC (together with SN and SP), which is more informative under severe class imbalance.

**Table 1:** The performance of PRIME-BSPre versus other methods on different cell lines $SN=\frac{TP}{FN+TP},\:SP=\frac{TN}{TN+FP},\:ACC=\frac{TN+TP}{TN+FP+FN+TP},MCC=\frac{TN\times\:TP-FN\times\:FP}{\surd\:\left(TN+FP\right)\left(FN+TP\right)\left(TN+FN\right)\left(TP+FP\right)}$ , where TP, TN, FN, FP are denoted as true positive, true negative, false negative and false positive in prediction results respectively

Methods	Cell lines	Performance			
		SN	SP	ACC	MCC
PRIME-BSPre	HepG2	0.72	0.75	0.75	**0.50**
	HEK293	0.59	0.75	0.72	**0.41**
PRIME-3D2D	HepG2	0.47	0.95	0.90	0.42
	HEK293	0.28	0.95	0.92	0.20
PrismNet	HepG2	0.17	0.95	0.89	0.08
	HEK293	0.02	0.99	0.95	0.01

To further quantify the contributions of the three components emphasized in the manuscript, we conducted a strict ablation study (Methods; Supplementary Table S3). Removing LS-PEAK produced the largest degradation in Top-1 MCC (mean MCC: 0.434 in FULL vs. 0.200 in –LS-PEAK), and nearly halved the proportion of high-quality predictions (Top-1 MCC > 0.6: 37.0% vs. 18.3%). Removing LS-seq also substantially reduced performance (mean MCC: 0.303; Top-1 MCC > 0.6: 20.8%). In contrast, removing LS-2d showed a small effect on the global mean (0.432 vs. 0.434 in FULL) but led to marked changes for a subset of RBP–RNA pairs, consistent with LS-2d acting as a complementary local-structure signal within LS-Score rather than the dominant driver across all cases.

### Low-entropy motifs capture cohort-level sequence, structure, and positional preferences of RBP binding

Across the template-library binding regions, the extracted low-entropy motifs exhibit a consistent sequence composition bias and an enriched secondary-structure context at the cohort level, indicating that low-entropy motifs provide an explicit summary of RBP binding preferences in both sequence and structure. In addition, transcript-wide normalization into 10 relative bins shows that most RBPs display broadly distributed binding, whereas a subset presents non-uniform positional enrichment along transcripts. These preference summaries are shown in Supplementary Fig. S1.

### A large-scale test on PRIME-BSPre and comparison with other methods

**Fig. 4:**
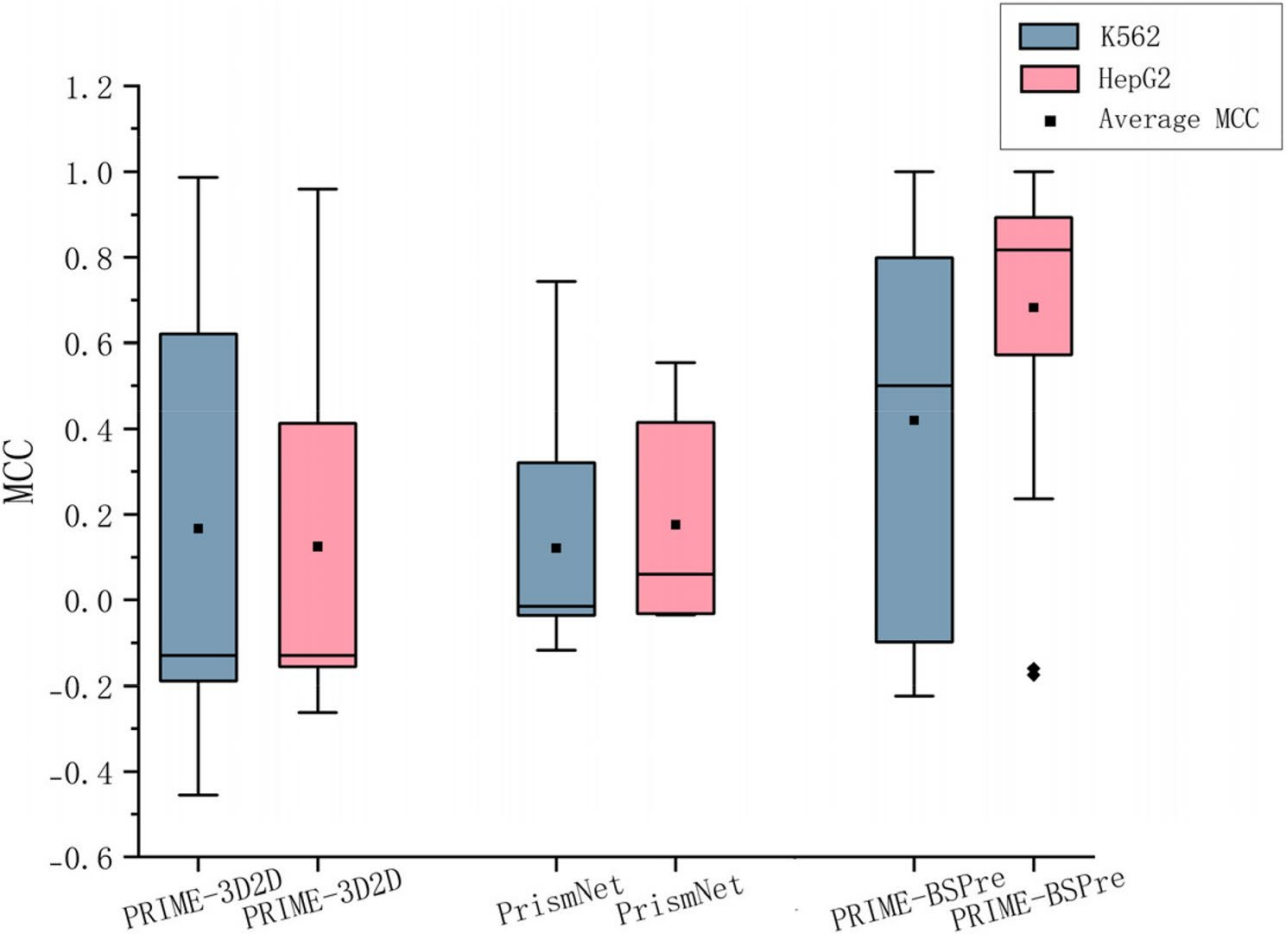
Prediction results compared with other methods on different cell lines. MCC of the prediction results of PRIME-BSPre, compared with PRIME-3D2D, PrismNet on the cell line K562 and the cell line HepG2 over 158 RBP-RNA test pairs. The box line plot shows overall results on different methods

Benchmark on Test set 2 demonstrates the PRIME-BSPre prediction ability for different cell lines when considering the binding strength of RBPs. Figure 4 shows the performance of PRIME-BSPre by using MCC as the main indicator. PRIME-3D2D and PrismNet are also tested on Test set 2. Taken together, PRIME-BSPre gives the best prediction performance, the average MCC on two cell lines of PRIME-BSPre predictions is 0.47, better than the results of PRIME-3D2D and PrismNet, and the performance on each cell line is also higher than other two methods.

### Compare PRIME-BSPre with PrismNet to demonstrate its applicability across diverse RBPs and cell lines

**Fig. 5:**
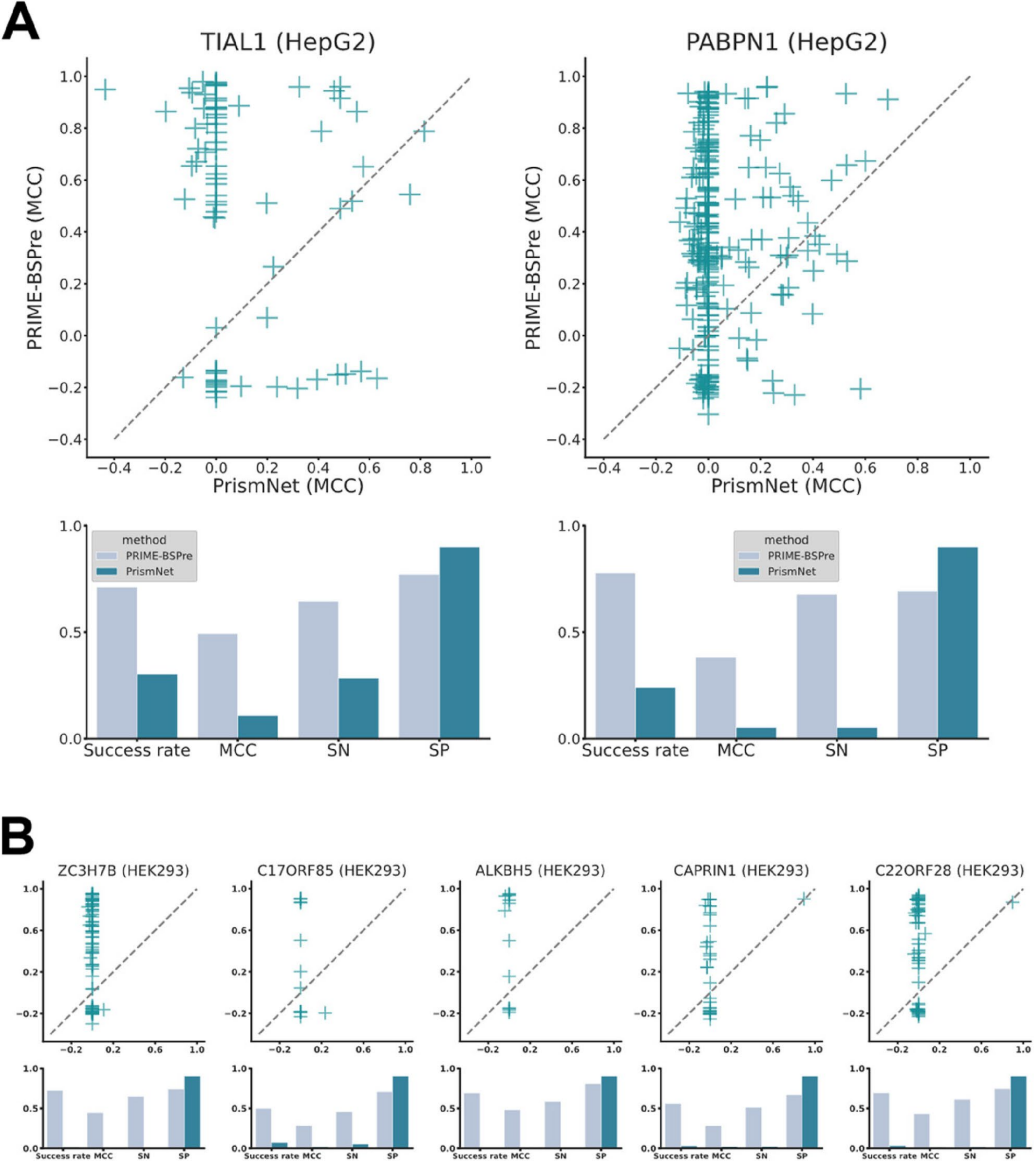
Comparison with PrismNet on different cell lines. **A-B** Comparison of PRIME-BSPre with PrismNet in different cell lines. Dot plots depict the performance of all RNAs in each RBP, as predicted by PRIME-BSPre and PrismNet

A comparison across different RBPs from several cell lines is conducted between PrismNet and PRIME-BSPre. PrismNet outputs used here were derived from the “Pre-calculated RBP binding sites” provided by its web server. To ensure objectivity in our comparison, CLIP data on the HepG2 cell line [7] and the HEK293 cell line [33] are selected for testing, as a significant proportion of the templates in PRIME-BSPre library were derived from the K562 cell line. While about PrismNet, the sequence & structure models trained on the HepG2 cell line are chosen. TIAL1 dataset (containing 76 RNAs) and PABPN1 dataset (containing 238 RNAs) were chosen as RBPs for comparison on the HepG2 cell line (Fig. 5A). It is noteworthy that the high specificity observed in both methods (Fig. 5A-B) can be attributed to the significantly shorter predicted binding sites compared to the full-length of the RNAs. This results in a high true negative rate for all predictions, so the validation of prediction requires to reach both high sensitivity (SN) and specificity (SP) simultaneously. Subsequently, RNA binding sites data from five RBPs (ZC3H7B, C17ORF85, ALKBH5, CAPRIN1, C22ORF28) obtained through PAR-CLIP experiments on the HEK293 cell line conducted by Baltz et al. [33] are utilized to compare the predictive performance of the two methods across different cell lines. The PrismNet’s model used to predict was also trained on the HepG2 cell line and PRIME-BSPre still relied on the original template library.

The utilization of deep learning methods for predicting RNA binding sites, as exemplified by PrismNet, still faces numerous challenges. Firstly, each RBP requires a distinct model to be trained, which consequently necessitates data on protein-RNA interactions. This raises the threshold as predictions are often made solely due to inadequate experimental data. Additionally, a model trained on one cell line is not able to transfer to other cell lines well in aforementioned comparison. The model seems to have acquired distinct binding preferences for each cell line or may have been influenced by the experimental conditions. In summary, although models trained using deep learning methods can accomplish some prediction tasks, they are far less stable and portable than template-based methods.

## Discussion

PRIME-BSPre is a computational method that predicts RNA binding sites through alignment against the template library, considering sequence similarity, secondary structure similarity, and motifs binding preference. Compared to current deep learning methods, PRIME-BSPre overcomes the challenge of requiring experimental data for training before prediction and exhibits greater robustness in predicting performance across different cell lines. Our benchmarks were conducted on full-length coding sequences (CDS) of protein-coding transcripts rather than short artificial fragments flanked by specific lengths. Additionally, we are the first to introduce a low Shannon entropy algorithm (see Materials and methods for parameterization and rationale). In the analysis of the features of low entropy motifs on template binding regions, we demonstrated the effectiveness of low entropy algorithm to select RBP binding motifs. Such selection method highlights the biological binding preferences of RBPs. The predicted results also support the notion that RBPs are more likely to bind low-complexity RNA motifs.

We acknowledge that restricting templates to CDS regions and imposing a maximum transcript length of 2000 nt limits the representativeness of the template library, particularly for long coding transcripts and for binding occurring in UTR or noncoding regions. However, this constraint was introduced as a controlled trade-off to ensure tractable computation and consistent template processing, and all methods in our comparison are evaluated under the same library definition. Future work will extend the library to include additional transcript regions and longer templates as computational scalability allows. In addition, the current benchmark focuses on protein-coding mRNAs (CDS sequences) and does not include non-coding RNA biotypes such as lncRNAs, tRNAs, or rRNAs. Extending PRIME-BSPre to non-coding RNAs will require building corresponding transcript annotations and handling distinct sequence-length distributions and mapping ambiguities (e.g., multi-copy rRNA loci).

Given the genome-wide nature of our prediction, it is observed that there exist some binding sites located within low-complexity, simple-repeat-like sequence segments (e.g., short homopolymers or di-/tri-nucleotide repeats), which are prevalent throughout the human genome [40, 41]. Notably, some binding sites appear to be closely associated with these repetitive regions, while others occur between repeat areas or even coincide with them. Moreover, these repetitive regions also exhibit characteristics of low complexity, such as continuous repetition of double bases or repeats in the form of a triple or quadruple that initiates with a single base. Some templates in our library contain binding sites within the repeat region, which are represented in lowercase letters in the genome. The scoring of sequence similarity takes the capitalization of bases into consideration, thereby enabling the prediction of certain binding sites based on this feature. NF90, previously mentioned, exhibits multiple repetitive regions within its interacting RNA transcripts, some of which encompass binding sites. Investigating the intricate interplay between these repeat regions and RBPs holds substantial scientific significance.

Recent advances have also emphasized cellular/condition-aware prediction of RBP–RNA interactions, where models are trained on large collections of in vivo CLIP/eCLIP datasets across multiple cellular contexts and may incorporate condition-matched covariates to capture dynamic binding behaviors (e.g., HDRNet, Reformer, and iDeepB) [42, 43]. These frameworks typically aim at single-nucleotide binding affinity or binding-profile (sequence-to-signal) prediction, and their performance is tightly coupled to the availability of matched training labels and, in some cases, matched auxiliary data (such as condition-specific RNA-seq or in vivo structure profiles) [43]. In contrast, PRIME-BSPre is positioned as a template-based, training-free and portable approach that outputs discrete binding-site calls through alignment to a fixed template library and LS-Score/LS-PEAK selection, enabling application when condition-matched training resources are limited or heterogeneous. Therefore, we did not include these condition-aware methods in our head-to-head benchmarking cohort, because a fair comparison would require matched multi-omics inputs for all test transcripts/cell lines and an additional, method-specific procedure to convert continuous profile outputs into discrete binding-site masks under identical data splits. We consider these approaches complementary rather than directly interchangeable, and integrating condition-specific signals into a template-based framework is an important direction for future work.

## Conclusion

PRIME-BSPre (Protein-RNA Interaction Modeling on Binding Sites Prediction) is a template-based method considering local similarity scoring to predict binding sites for protein RNA interactions. Experimental evidence suggests that RBPs exhibit a conservative preference for specific RNA motifs, with a particular affinity for low complexity motifs [23]. Additionally, RNA secondary structure is also crucial in protein-RNA interactions [23, 44, 45]. Compared to the aforementioned methods that directly learn binding preferences for predicting binding sites, our template-based approach offers the advantage of utilizing native binding sites in preliminary alignment, considering sequence and secondary structure similarities. Besides, tertiary structural similarity between RBPs was also taken into account [38]. We propose that when the target and template exhibit high structure and sequence similarity, their binding preferences are likely to be similar as well. And the alignment also revealed that around 6% of the templates exhibited efficacy (MCC > 0.6) in each prediction. The assessment of binding preference similarity between templates and targets incorporates the complexity of RNA motifs by calculating the Shannon entropy of RNA sequences within an 8-mer sliding window. Moreover, we have devised a novel LS-2d approach for quantifying secondary structures, enabling the comparison of characteristic structural features between templates and targets. Compared with the state-of-art deep learning method, PRIME-BSPre performed better in predicting binding sites across different cell lines.

## Supplementary Information

Supplementary Material 1.

Supplementary Material 2.

Supplementary Material 3.

Supplementary Material 4.

Supplementary Material 5.

Supplementary Material 6.

## Data Availability

All the comparison datasets and their prediction performances have been uploaded at [http://rnabinding.com/dataset_performance.rar].

## Declarations

### Code availability

Code is available under http://rnabinding.com/PRIME-BSPre.tar.gz.

## References

[1] England WE, et al. An atlas of posttranslational modifications on RNA binding proteins. Nucleic Acids Res. 2022;50:4329–39. 35438783 10.1093/nar/gkac243 PMC9071496

[2] Spitale RC, et al. Structural imprints in vivo Decode RNA regulatory mechanisms. Nature. 2015;519:486–90. 25799993 10.1038/nature14263 PMC4376618

[3] Van Nostrand EL, et al. A large-scale binding and functional map of human RNA-binding proteins. Nature. 2020;583:711–9. 32728246 10.1038/s41586-020-2077-3 PMC7410833

[4] Van Nostrand EL, et al. Robust transcriptome-wide discovery of RNA-binding protein binding sites with enhanced CLIP (eCLIP). Nat Methods. 2016;13:508–14. 27018577 10.1038/nmeth.3810 PMC4887338

[5] Gerstberger S, Hafner M, Tuschl T. A census of human RNA-binding proteins. Nat Rev Genet. 2014;15:829–45. 25365966 10.1038/nrg3813 PMC11148870

[6] Kuret, et al. Positional motif analysis reveals the extent of specificity of protein-RNA interactions observed by CLIP. Genome Biol. 2022;23:191. 36085079 10.1186/s13059-022-02755-2 PMC9461102

[7] Xu Y, et al. PrismNet: predicting protein-RNA interaction using in vivo RNA structural information. Nucleic Acids Res. 2023;51:W468–77. 10.1093/nar/gkad353 PMC10320048 37140045

[8] Laverty KU, et al. PRIESSTESS: interpretable, high-performing models of the sequence and structure preferences of RNA-binding proteins. Nucleic Acids Res. 2022;50:e111. 36018788 10.1093/nar/gkac694 PMC9638913

[9] Konig J, et al. iCLIP reveals the function of HnRNP particles in splicing at individual nucleotide resolution. Nat Struct Mol Biol. 2010;17:909–15. 20601959 10.1038/nsmb.1838 PMC3000544

[10] Hafner M, et al. Transcriptome-wide identification of RNA-binding protein and MicroRNA target sites by PAR-CLIP. Cell. 2010;141:129–41. 20371350 10.1016/j.cell.2010.03.009 PMC2861495

[11] Cook KB, Hughes TR, Morris QD. High-throughput characterization of protein-RNA interactions. Brief Funct Genomics. 2015;14:74–89. 25504152 10.1093/bfgp/elu047 PMC4303715

[12] Cook KB, et al. RNAcompete-S: combined RNA sequence/structure preferences for RNA binding proteins derived from a single-step in vitro selection. Methods. 2017;126:18–28. 28651966 10.1016/j.ymeth.2017.06.024

[13] Lambert N, et al. RNA Bind-n-Seq: quantitative assessment of the sequence and structural binding specificity of RNA binding proteins. Mol Cell. 2014;54:887–900. 24837674 10.1016/j.molcel.2014.04.016 PMC4142047

[14] Zheng J, Hong X, Xie J, Tong X, Liu S. P3DOCK: a protein–RNA Docking webserver based on template-based and template-free Docking. Bioinformatics. 2020;36(1):96–103. 31173056 10.1093/bioinformatics/btz478

[15] Zheng J, Kundrotas PJ, Vakser IA, Liu S. Template-Based modeling of Protein-RNA interactions. PLoS Comput Biol. 2016;12(9):e1005120. 27662342 10.1371/journal.pcbi.1005120 PMC5035060

[16] Orenstein Y, Wang Y, Berger B. RCK: accurate and efficient inference of sequence- and structure-based protein-RNA binding models from RNAcompete data. Bioinformatics. 2016;32:i351–9. 27307637 10.1093/bioinformatics/btw259 PMC4908343

[17] Maticzka D, Lange SJ, Costa F, Backofen R. GraphProt: modeling binding preferences of RNA-binding proteins. Genome Biol. 2014;15:R17. 24451197 10.1186/gb-2014-15-1-r17 PMC4053806

[18] Kazan H, Ray D, Chan ET, Hughes TR, Morris Q. RNAcontext: a new method for learning the sequence and structure binding preferences of RNA-binding proteins. PLoS Comput Biol. 2010;6:e1000832. 20617199 10.1371/journal.pcbi.1000832 PMC2895634

[19] Ding Y, Lawrence CE. A statistical sampling algorithm for RNA secondary structure prediction. Nucleic Acids Res. 2003;31:7280–301. 14654704 10.1093/nar/gkg938 PMC297010

[20] Spitale R, Flynn R, Zhang Q, et al. Structural imprints in vivo Decode RNA regulatory mechanisms. Nature. 2015;519:486–90. 25799993 10.1038/nature14263 PMC4376618

[21] Alipanahi B, Delong A, Weirauch MT, Frey BJ. Predicting the sequence specificities of DNA- and RNA-binding proteins by deep learning. Nat Biotechnol. 2015;33:831–8. 26213851 10.1038/nbt.3300

[22] Hiller M, Pudimat R, Busch A, Backofen R. Using RNA secondary structures to guide sequence motif finding towards single-stranded regions. Nucleic Acids Res. 2006;34:e117. 16987907 10.1093/nar/gkl544 PMC1903381

[23] Dominguez D, et al. Sequence, Structure, and context preferences of human RNA binding proteins. Mol Cell. 2018;70:854–e867859. 29883606 10.1016/j.molcel.2018.05.001 PMC6062212

[24] Ray D, et al. A compendium of RNA-binding motifs for decoding gene regulation. Nature. 2013;499:172–7. 23846655 10.1038/nature12311 PMC3929597

[25] Xie J, Zheng J, Hong X, Tong X, Liu S. PRIME-3D2D is a 3D2D model to predict binding sites of protein-RNA interaction. Commun Biol. 2020;3:384. 32678300 10.1038/s42003-020-1114-y PMC7366699

[26] Luo Y, et al. New developments on the encyclopedia of DNA elements (ENCODE) data portal. Nucleic Acids Res. 2020;48:D882–9. 31713622 10.1093/nar/gkz1062 PMC7061942

[27] Cunningham F, et al. Ensembl 2022. Nucleic Acids Res. 2022;50:D988–95. 34791404 10.1093/nar/gkab1049 PMC8728283

[28] Danecek P, et al. Twelve years of samtools and BCFtools. Gigascience. 2021;10:giab008. 10.1093/gigascience/giab008 PMC7931819 33590861

[29] Lander ES, et al. Initial sequencing and analysis of the human genome. Nature. 2001;409:860–921. 11237011 10.1038/35057062

[30] Reuter JS, Mathews DH. RNAstructure: software for RNA secondary structure prediction and analysis. BMC Bioinformatics. 2010;11:129. 20230624 10.1186/1471-2105-11-129 PMC2984261

[31] Varadi M, et al. AlphaFold protein structure database: massively expanding the structural coverage of protein-sequence space with high-accuracy models. Nucleic Acids Res. 2022;50:D439–44. 34791371 10.1093/nar/gkab1061 PMC8728224

[32] Jumper J, et al. Highly accurate protein structure prediction with alphafold. Nature. 2021;596:583–9. 34265844 10.1038/s41586-021-03819-2 PMC8371605

[33] Baltz AG, et al. The mRNA-bound proteome and its global occupancy profile on protein-coding transcripts. Mol Cell. 2012;46:674–90. 22681889 10.1016/j.molcel.2012.05.021

[34] Lodde V, et al. Systematic identification of NF90 target RNAs by iCLIP analysis. Sci Rep. 2022;12:364. 35013429 10.1038/s41598-021-04101-1 PMC8748789

[35] Blue SM, et al. Transcriptome-wide identification of RNA-binding protein binding sites using seCLIP-seq. Nat Protoc. 2022;17:1223–65. 35322209 10.1038/s41596-022-00680-z PMC11134598

[36] Li Q, et al. Measuring reproducibility of high-throughput experiments. Annals Appl Stat. 2011;5:1752–79.

[37] Xinzhou Ge. et al. Clipper: p-value-free FDR control on high-throughput data from two conditions. Genome Biol. 2021;22:288. 10.1186/s13059-021-02506-9 PMC8504070 34635147

[38] Zhang Y, Skolnick J. TM-align: a protein structure alignment algorithm based on the TM-score. Nucleic Acids Res. 2005;33:2302–9. 15849316 10.1093/nar/gki524 PMC1084323

[39] Will S, Joshi T, Hofacker IL, Stadler PF, Backofen R. LocARNA-P: accurate boundary prediction and improved detection of structural RNAs. RNA. 2012;18:900–14. 22450757 10.1261/rna.029041.111 PMC3334699

[40] Gemmell NJ, Repetitive DNA. Genomic dark matter matters. Nat Rev Genet. 2021;22:342. 33782602 10.1038/s41576-021-00354-8

[41] de Koning AP, Gu W, Castoe TA, Batzer MA, Pollock DD. Repetitive elements May comprise over two-thirds of the human genome. PLoS Genet. 2011;7:e1002384. 22144907 10.1371/journal.pgen.1002384 PMC3228813

[42] Zhu H, et al. Dynamic characterization and interpretation for protein-RNA interactions across diverse cellular conditions using HDRNet. Nat Commun. 2023;14:6824. 37884495 10.1038/s41467-023-42547-1 PMC10603054

[43] Shen X, et al. A deep learning model for characterizing protein-RNA interactions from sequences at single-base resolution. Patterns (N Y). 2025;6:101150. 39896261 10.1016/j.patter.2024.101150 PMC11783876

[44] Sun L, et al. Predicting dynamic cellular protein-RNA interactions by deep learning using in vivo RNA structures. Cell Res. 2021;31:495–516. 33623109 10.1038/s41422-021-00476-y PMC7900654

[45] Stefl R, Skrisovska L, Allain FH. RNA sequence- and shape-dependent recognition by proteins in the ribonucleoprotein particle. EMBO Rep. 2005;6:33–8. 15643449 10.1038/sj.embor.7400325 PMC1299235

