# Supplementary material for: PRIME-BSPre: a genome-wide protein-RNA binding sites prediction method based on templates
Source: BMC Genomics. 2026 Feb 21;27:317. doi: 10.1186/s12864-026-12657-3 (PMC13032426; doi:10.1186/s12864-026-12657-3)
Supplement: Supplementary file 1 — Supplementary Material 1. [file 12864_2026_12657_MOESM1_ESM.pdf]

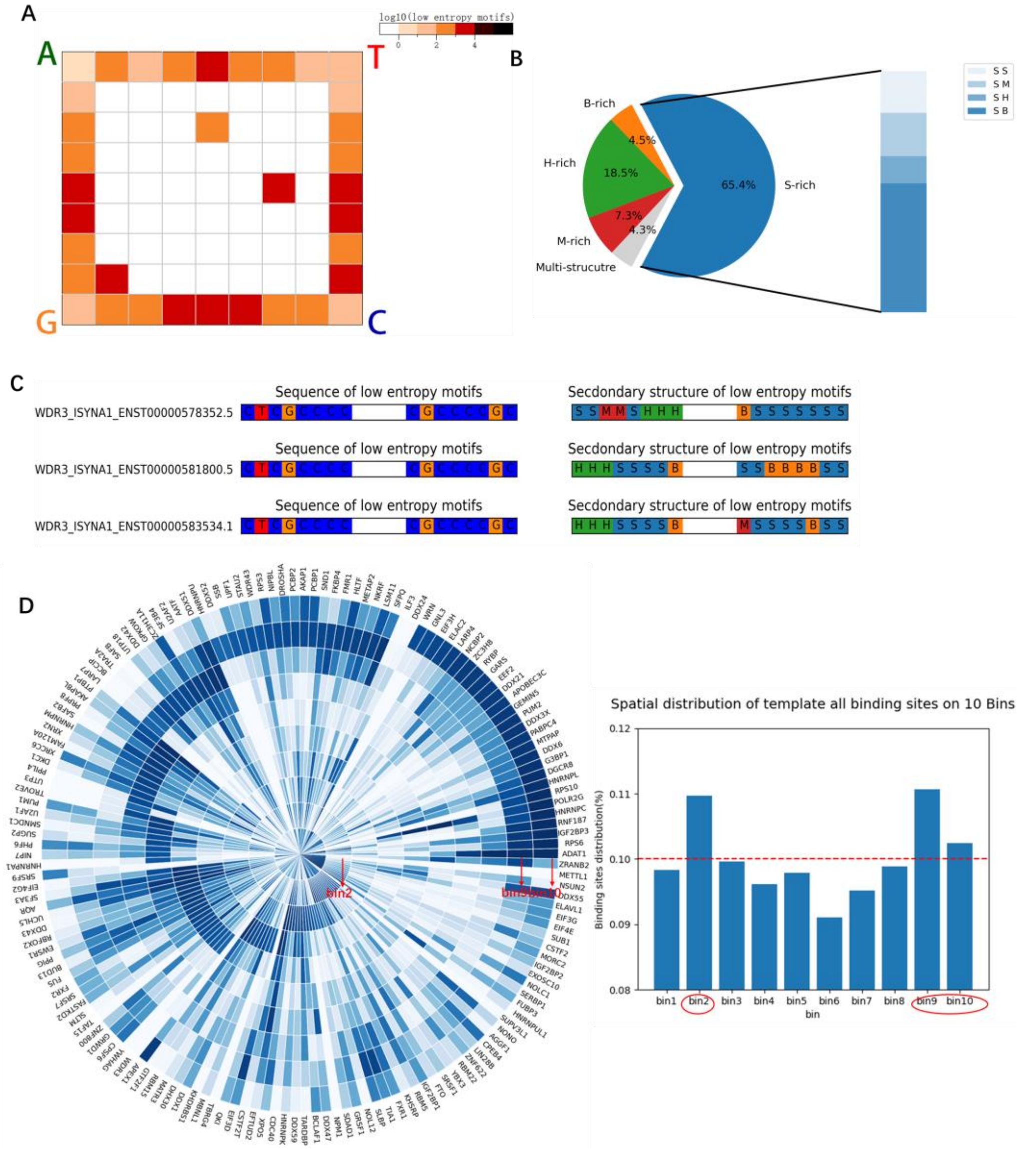

Fig. S1 Features of low entropy motifs of the template library binding regions. We used a low entropy algorithm to select low entropy motifs in the binding regions of the template library and analyzed the sequence and secondary structure distribution features of these motifs. In addition, we analyzed the binding preference on the spatial location of template RBPs. **A** Base distribution of low entropy motifs on template binding region. **B** Secondary structure distribution of low entropy motifs on template binding region. **C** Example of distribution map with base and secondary structure on AATF_GP1BB_ENST00000366425.4 low entropy motifs. **D** Spatial distribution of the template binding regions on 10 bins.

Low-entropy motif screening and binding-preference summarization

Methods

Low-entropy motif extraction. Low-entropy motifs were extracted from template-library binding regions using Shannon entropy computed on a sliding window. For each binding-region sequence, we scanned an 8-nt window with a 6-nt step. For a window with nucleotide frequencies pA, pC, pG, pU (computed within the 8-nt window), Shannon entropy was calculated as:

$$H = - \Sigma\_\{b \in \{A, C, G, U\}\} p\_b \cdot log2(p\_b).$$

Windows with $H < 1.1$ were retained as low-entropy motifs (Supplementary Table S2). When multiple low-entropy windows overlapped, all retained windows were included in downstream summaries (i.e., no additional merging/deduplication was applied).

(A) Sequence preference summary (Supplementary Fig. S1A). To summarize sequence-level preferences, we aggregated nucleotide composition across all retained low-entropy motifs in the template library. Specifically, we computed the overall base fractions:

f_b = (count of base b across all retained motifs) / (total nucleotides across all retained motifs), for b ∈ {A, C, G, U}.

We report the resulting global base-composition enrichment as Supplementary Fig. S1A.

(B-C) Secondary-structure preference summary (Supplementary Fig. S1B). For each retained low-entropy motif, we obtained its predicted RNA secondary-structure string and converted it into five coarse structural units: B (bulge), E (external strand), H (hairpin loop), M (multi-loop), and S (stem). For a motif of length 8, let L_u denote the number of positions assigned to unit u ∈ {B, E, H, M, S}. A motif was labeled u-rich if L_u ≥ 4 (i.e., at least half of the motif length); if no unit satisfied this criterion, the motif was labeled multi-structure. We then quantified the distribution of these motif categories across all retained low-entropy motifs and report it as Supplementary Fig. S1B.

(D) Positional binding preference along transcripts (Supplementary Fig. S1D). To assess positional preference, each transcript in the template library was normalized into 10 equal-length relative bins from 5′ to 3′. For each binding site, its location was mapped to one of the 10 bins; counts were then aggregated to obtain the overall bin-wise distribution, normalized by the total number of binding sites. The resulting positional distribution is shown in Supplementary Fig. S1D.
